# Health Literacy Association With Health Behaviors and Health Care Utilization in Multiple Sclerosis: A Cross-Sectional Study

**DOI:** 10.2196/ijmr.2993

**Published:** 2014-02-10

**Authors:** Ruth Ann Marrie, Amber Salter, Tuula Tyry, Robert J Fox, Gary R Cutter

**Affiliations:** ^1^University of ManitobaDepartments of Internal Medicine and Community Health SciencesWinnipeg, MBCanada; ^2^University of Alabama at BirminghamDepartment of BiostatisticsBirmingham, ALUnited States; ^3^Barrow Neurological InstituteDivision of NeurologyPhoenix, AZUnited States; ^4^Neurological InstituteMellen Center for Multiple SclerosisCleveland ClinicCleveland, OHUnited States

**Keywords:** multiple sclerosis, health literacy, health care utilization, comorbidity, health behaviors

## Abstract

**Background:**

Low health literacy is generally associated with poor health outcomes; however, health literacy has received little attention in multiple sclerosis (MS).

**Objective:**

The aim of this study was to investigate the health literacy of persons with MS using the North American Research Committee on Multiple Sclerosis (NARCOMS) Registry.

**Methods:**

In 2012, we conducted a cross-sectional study of health literacy among NARCOMS participants. Respondents completed the Medical Term Recognition Test (METER) which assesses the ability to distinguish medical and nonmedical words, and the Newest Vital Sign (NVS) instrument which evaluates reading, interpretation, and numeracy skills. Respondents reported their sociodemographic characteristics, health behaviors, comorbidities, visits to the emergency room (ER), and hospitalizations in the last 6 months. We used logistic regression to evaluate the characteristics associated with functional literacy, and the association between functional literacy and health care utilization.

**Results:**

Of 13,020 eligible participants, 8934 (68.6%) completed the questionnaire and were US residents. Most of them performed well on the instruments with 81.04% (7066/8719) having functional literacy on the METER and 74.62% (6666/8933) having adequate literacy on the NVS. Low literacy on the METER or the NVS was associated with smoking, being overweight or obese (all *P*<.001). After adjustment, low literacy on the METER was associated with ER visits (OR 1.28, 95% CI 1.10-1.48) and hospitalizations (OR 1.19, 95% CI 0.98-1.44). Findings were similar for the NVS.

**Conclusions:**

In the NARCOMS cohort, functional health literacy is high. However, lower levels of health literacy are associated with adverse health behaviors and greater health care utilization.

## Introduction

The elements of general literacy include the knowledge and skills to comprehend and use written information, to locate and use information captured in documents such as maps, and numeracy. Health literacy builds on these concepts [1], and refers to the capacity of individuals to gather, process, and comprehend the basic health information and services needed to support health-related decision making. Individuals need to be able to understand written health information and to communicate verbally about health, so that they can make decisions about health promotion, health protection, disease prevention, health care maintenance, and to navigate the health care system [2].

A growing literature suggests that lower health literacy is associated with higher rates of health care utilization and mortality, lower rates of health promoting activities, lower adherence to therapy and less successful disease control [1,3-7]. Despite this recognition in other populations, and the frequent interactions with the health system required by affected people [8,9], the issue of health literacy has received little attention in multiple sclerosis (MS) population [10].

We aimed to investigate the health literacy of persons with MS in a sociodemographically diverse population from the United States, and to estimate the associations between health literacy and health behaviors, comorbidities, and health care utilization. We hypothesized that lower health literacy would be associated with a higher frequency of smoking, obesity, and greater health care utilization.

## Methods

### North American Research Committee on Multiple Sclerosis Registry

The North American Research Committee on Multiple Sclerosis (NARCOMS) Registry is a voluntary self-report registry for people with MS, developed by the Consortium of MS Centers [11]. We have validated diagnoses of MS in a randomly selected sample of participants [12]. NARCOMS participants agree to the use of their de-identified data for research purposes, and the Registry is approved by the Institutional Review Board at the University of Alabama at Birmingham.

Participants may enroll by completing a questionnaire online, or by mailing in a questionnaire [11]. After enrollment, participants are asked to complete surveys semi-annually, on paper or online per their preference. On each survey participants report sociodemographic and clinical information, including disability status using Patient Determined Disease Steps (PDDS) and Performance Scales (PS) [13,14]. The PDDS is a validated measure which correlates highly with a physician-scored Expanded Disability Status Scale (EDSS) [13,15]. It is scored ordinally from 0 to 8, where a score of 0 approximates an EDSS score of 0, a score of 3 represents early gait disability without needing an assistive device and approximates an EDSS score of 4.0 to 4.5; and scores of 4, 5, and 6 represent EDSS scores of 6.0 to 6.5. PS uses a single question to assess eight domains, including mobility, bowel/bladder, fatigue, sensory, vision, cognition, spasticity, and hand [14]. All of the subscales are scored as follows: 0 (normal), 1 (minimal), 2 (mild), 3 (moderate), 4 (severe), or 5 (total disability), except mobility which is scored from 0 to 6. The cognition subscale correlates strongly with the Perceived Deficits Questionnaire (*r*=.71, *P*<.001) [13], a 20-item self-reported questionnaire for cognition incorporated in the Multiple Sclerosis Quality of Life Inventory [16]. Construct validity of the cognitive subscale is supported by moderate correlations (*r*=.70, *P*<.001) with the Modified Fatigue Impact Scale (convergent validity) but not with age (*r*=.11, *P*=.46, divergent validity) [13].

Participants report the presence or absence of specific comorbidities using the following question format “Has a doctor ever told you that you have…?”[17]. We have previously shown the validity of our self-reported comorbidity questionnaire [18]. Based on our prior work, the comorbidities of interest were diabetes, hypertension, hyperlipidemia, heart disease, migraine, irritable bowel syndrome, chronic lung disease, cancer, obstructive sleep apnea, autoimmune thyroid disease, depression, and anxiety [19,20].

Current smoking status is assessed using a validated question from the Behavioral Risk Factor Surveillance System, and reported as none, some days or every day [21]. We assess the frequency of alcohol intake in the prior 6 months with the first question from the AUDIT-C, a screening instrument developed to identify persons with recent heavy drinking and alcohol dependence [22]. Responses are never, monthly or less, two to four times a month, three to four times a week, and four or more times a week. Body mass index (BMI) is calculated from self-reported height and weight. Overweight is defined as BMI≥25 and BMI<30, and obesity as BMI≥30 [23].

With respect to health care utilization in the last 6 months, participants report whether they had any visits to an emergency room (ER), and whether they were hospitalized overnight.

### Health Literacy

We asked NARCOMS participants about health literacy in 2012. Multiple generic health literacy instruments have been developed [24]. We selected three instruments validated in other populations based on several considerations. First, no instrument fully captures the construct of health literacy as defined by a person’s ability to seek, understand, and use health information [24]; thus multiple instruments were needed. Second, we selected instruments that were brief and easy to administer to minimize participant burden. We used the eHealth Literacy Scale (eHEALS) which includes eight items that assess the knowledge, comfort and perceived skills of persons completing the scale, who are seeking and using electronic health information to address health concerns. It also includes two optional questions designed to assess the participant’s interest in using e-health tools. The instrument was developed based on social cognitive and self-efficacy theory. It has been validated and showed good internal consistency (alpha=.88) and test-retest reliability [25].

The Medical Term Recognition Test (METER) is a brief, self-administered questionnaire that was developed to address other instruments’ limitations such as excessive length, or the requirement that a practitioner administers the tool. The METER is composed of 40 medical words and 30 nonwords [26]. The respondent is asked to mark the words they recognize as actual words, and the METER is scored as the number of correctly identified words minus the number of incorrectly identified words. According to the developers, the format of the instrument was based on tests such as the Author Recognition Test and other similar tests which correlate highly with measures of vocabulary, reading comprehension, and verbal fluency. Scores of 0-20 indicate low literacy, 21-34 indicate marginal literacy, and 35-40 indicate functional literacy. The instrument has high internal consistency (alpha=.93), which correlates highly with the Rapid Estimate of Adult Literacy in Medicine (REALM) questionnaire (*r*=.74), an interviewer-administered measure of literacy, and is associated with cardiovascular health.

The Newest Vital Sign (NVS) Instrument is a nutrition label from an ice cream container that is accompanied by six questions aimed to test reading, interpretation, and numeracy skills [27]. For example one question asks how many calories would be consumed if an entire container were eaten, while another asks whether it is safe for a person with peanut allergy to eat the ice cream. One point is scored for each correct answer. Scores from 0-1 suggest a high likelihood of marginal or inadequate literacy; 2-3 suggest possible marginal or inadequate literacy, while scores of 4-6 indicate adequate literacy. The NVS requires about three minutes for administration. Scores of less than 4 suggest low health literacy. The internal consistency of the instrument is good (alpha=.76), and it has good criterion validity as compared to the Test of Functional Health Literacy in Adults (TOFLHA).

### Analysis

We restricted our analysis to NARCOMS participants living in the United States. Missing responses were not imputed. Performance on each of the instruments was scored as described above.

Given that the three instruments used to assess health literacy have not been used in the MS population previously, we also report the internal consistency for the two multi-item instruments (NVS, eHEALS) as measured using Cronbach’s alpha [28]. We summarized categorical variables using frequency (percent [%]), and continuous variables using mean (standard deviation [SD]) or median (interquartile range [IQR]) as appropriate.

After categorizing the scores for the METER and NVS as described above, we estimated the associations between health literacy and health behaviors, comorbidity, and health care utilization. Health behaviors included current smoking (yes vs no), overweight or obesity versus normal weight, any alcohol intake (yes vs no). Comorbidity was evaluated as any comorbidity versus no comorbidity, and as the number of comorbidities. Health care utilization included ER visits (yes vs no) and hospitalizations (yes vs no). Univariate analyses employed chi-square tests.

Multivariable analyses employed binary logistic regression. For these analyses we dichotomized the METER at the cutpoint for functional literacy (≤34 [low literacy] vs >35 [functional literacy]), and the NVS at the cutpoint for adequate literacy (<4 [low literacy] vs ≥4 [adequate literacy]). First we evaluated the association between participant characteristics and having functional/adequate literacy. We constructed separate models for the METER and the NVS. Second, we separately modeled the association of health literacy with the outcomes of any ER visits and any hospitalizations. The independent variables considered for each regression model are described below.

### Covariates

For gender, female was the reference category. Race was categorized as white (reference group), and nonwhite. Education was included as indicator variables for high school diploma or less (reference group), Associate’s Degree or Technical Degree, Bachelor’s Degree, and post-graduate degree. Annual household income was included as indicator variables for <$15,000 (reference group), $15,000-29,999, $30,000-49,999, $50,000-100,000, and >$100,000, or declined to answer. Insurance status was included as indicator variables for private, public only (reference group), or none. Age was categorized as ≤35 (reference group), >35 to ≤50, >50 to ≤65, and >65 years. Using PDDS, participants were classified as having mild (0-2), moderate (3-4), or severe (5-8, reference group) disability [29]. Using PS cognition subscale, participants were classified as having normal (0, reference group), mildly impaired (1-2), or moderately to severely (3-5) impaired cognition.

Assumptions of models were tested using standard methods [30]. For each logistic regression model we used adjusted odds ratios (OR) and 95% confidence intervals (CI) as measures of association. We report a c-statistic as a measure of discriminating ability (estimate of area under the curve) and the Hosmer Lemeshow test as a measure of goodness of fit. Analyses were performed using SAS V9.2 (SAS Institute Inc, Cary, NC).

## Results

### Respondents

Of 13,020 eligible participants, 9019 (69.27%) completed the spring 2012 questionnaire. As compared to responders, nonresponders were more likely to be nonwhite (*P*<.001), to have a lower level of education (*P*<.001) and lower annual income (*P*=.007). They did not differ with respect to gender. Nonresponders were slightly younger (mean 53.70, SD 11.69) than responders (mean 57.02, SD 10.39, *P*<.001). Mean age at onset of MS symptoms was also younger in nonresponders (mean 30.12, SD 9.99) than responders (mean 30.97, SD 10.04, *P*<.001), but the difference of less than a year is unlikely to be clinically relevant. Of those who completed the questionnaire, 8934 (99.06%) were US residents and were included in this analysis. The demographic and clinical characteristics of the responders are summarized in Table 1.

**Table 1:** Characteristics of eligible responders to the NARCOMS Spring 2012 Questionnaire (n=8934^a^).

Characteristic			n (%) or mean (SD)
**Gender, n (%)**			
	Female		6984/8934 (78.17)
	Male		1950/8934 (21.83)
**Race, n (%)**			
	White		7818/8198 (95.36)
	Other		380/8198 (4.64)
**Education, n (%)**			
	High school diploma or less		2364/8792 (26.89)
	Associate’s/technical degree		1804/8792 (20.52)
	Bachelor’s degree		2258/8792 (29.09)
	Post-graduate degree		2066/8792 (23.50)
**Annual income, n (%)**			
	<$15,000		725/8753 (8.28)
	$15,000-29,999		1278/8753 (14.60)
	$30,000-49,999		1410/8753 (16.11)
	$50,000-100,000		2201/8753 (25.15)
	>$100,000		1315/8753 (15.02)
	I do not wish to answer		1824/8753 (20.84)
**Health insurance, n (%)**			
	Private		3869/7978 (48.50)
	Public only		3867/7978 (48.47)
	None		242/7978 (3.03)
Current age (years), mean (SD)			57.07 (10.39)
Age of symptom onset (years), mean (SD)			30.97 (10.04)
**Patient Determined Disease Steps, n (%)**			
	Mild (0-2)		3146/8845 (35.57)
	Moderate (3-4)		2301/8845 (26.01)
	Severe (5-8)		3398/8845 (38.42)
**Cognition, n (%)**			
	Normal		1969/8840 (22.3)
	Mild		4695/8840 (53.1)
	Moderate-severe		2176/8840 (24.6)

^a^The number of total responses for each characteristic varied as the respondents were not required to answer every question.

### Electronic Health Information and Health Literacy

The internal consistency reliability of the NVS was .74, and of the eHEALS was .94. Most respondents performed well on the health literacy instruments. On the METER, 1.84% (160/8719) had low literacy, 17.12% (1493/8719) had marginal literacy, and 81.04% (7066/8719) had functional literacy. On the NVS, 10.81% (966/8933) of respondents had a high likelihood of inadequate literacy, 14.56% (2267/8933) possibly had inadequate literacy, while 74.62% (6666/8933) had adequate literacy. Only 1.03% (90/8718) of participants had low literacy on both the METER and the NVS, while 65.52% (5712/8718) had functional literacy/numeracy on both instruments. METER scores correlated weakly with NVS scores (*r*=.31, *P*<.001).

The mean (SD) score on the eHEALS was 28.15 (18.57). eHEALS scores correlated quite weakly with scores on the METER (*r*=.14, *P*<.001) and the NVS (*r*=.24, *P*<.001). The mean (SD) eHEALS score was lower among persons with low literacy on the METER (mean 16.89, SD 16.86) than among persons with functional literacy (mean 29.24, SD 18.35, *P*<.001). Similarly, the mean (SD) eHEALS score was lower among persons with a high likelihood of inadequate literacy on the NVS (mean 17.36, SD 17.22) than among persons with adequate literacy (mean 30.40, SD 18.02, *P*<.001).

On univariate analysis, several sociodemographic characteristics were associated with functional health literacy (Tables 2 and 3). When assessed using the METER and the NVS, women, respondents with a higher level of education, higher level of income, and private health insurance were more likely to have functional literacy. Lower levels of self-reported disability and cognitive impairment, and shorter disease duration were also associated with an increased frequency of functional literacy based on the METER and the NVS.

Using multivariable logistic regression, gender (females), higher levels of education, higher levels of income, and older age, were associated with higher odds of having functional health literacy as assessed by the METER (Table 4). Higher levels of cognitive impairment were associated with lower odds of functional literacy. When literacy was assessed using the NVS, the findings were similar with the exception that higher levels of disability, as measured by the PDDS, were also associated with decreased odds of adequate literacy.

**Table 2:** Univariate associations between participant characteristics and health literacy as measured by the Medical Term Recognition Test (METER).

Characteristic^a^			METER			*P* value
			0-20	21-34	35-40	
**Gender, n (%), n=8719**						
	Female		109 (68.13)	1013 (67.85)	5708 (80.78)	<.001
	Male		51 (31.88)	480 (32.15)	1358 (19.22)	
**Race, n (%), n=7996**						
	White		139 (94.56)	1278 (94.53)	6211 (95.60)	.20
	Other		8 (5.44)	74 (5.5)	286 (4.4)	
**Education, n (%), n=8661**						
	High school diploma or less		67 (42.41)	534 (36.11)	1714 (24.40)	<.001
	Associate’s/technical degree		36 (22.78)	397 (26.84)	1345 (19.15)	
	Bachelor’s degree		30 (18.99)	355 (24.00)	2147 (30.57)	
	Post-graduate degree		25 (15.82)	193 (13.05)	1818 (25.88)	
**Annual income, n (%), n=8625**						
	<$15,000		31 (20.81)	171 (11.67)	497 (7.09)	<.001
	$15,000-29,999		33 (22.15)	257 (17.54)	966 (13.78)	
	$30,000-49,999		26 (17.45)	270 (18.43)	1106 (15.78)	
	$50,000-100,000		16 (10.74)	334 (22.80)	1829 (26.09)	
	>$100,000		15 (10.07)	129 (8.81)	1163 (16.59)	
	I do not wish to answer		28 (18.79)	304 (20.75)	1450 (20.68)	
**Health insurance, n (%), n=7780**						
	Private		36 (24.49)	526 (38.73)	3191 (50.85)	<.001
	Public only		97 (65.99)	777 (57.22)	2917 (46.49)	
	None		14 (9.52)	55 (4.05)	167 (2.66)	
Current age (years), mean (SD)			59.59 (10.19)	56.69 (10.86)	57.03 (10.26)	.007
Disease duration (years), mean (SD)			29.01 (12.16)	26.19 (12.00)	25.95 (11.97)	.007
**Patient Determined Disease Steps, n (%), n=8646**						
	Mild		37 (24.18)	469 (31.71)	2580 (36.78)	<.001
	Moderate		37 (24.18)	391 (26.44)	1831 (26.10)	
	Severe		79 (51.63)	619 (41.85)	2603 (37.11)	
**Cognition, n (%), n=8652**						
	Normal		28 (18.06)	278 (18.86)	1626 (23.15)	<.001
	Mild		73 (47.10)	771 (52.31)	3766 (53.62)	
	Moderate-severe		54 (34.84)	425 (28.83)	1631 (23.22)	

^a^The number of total responses for each characteristic varied as the respondents were not required to answer every question.

**Table 3:** Univariate associations between participant characteristics and health literacy as measured by the Newest Vital Sign (NVS).

Characteristic^a^			NVS			*P* value
			0-1	2-3	4-6	
**Gender, n (%), n=8933**						
	Female		654 (67.70)	926 (71.18)	5404 (81.07)	<.001
	Male		312 (32.30)	375 (28.82)	1262 (18.93)	
**Race, n (%), n=8197**						
	White		861 (94.93)	1124 (93.74)	5832 (95.75)	.0085
	Other		46 (5.07)	75 (6.26)	259 (4.25)	
**Education, n (%), n=8791**						
	High school diploma or less		357 (40.99)	457 (35.56)	1550 (23.36)	<.001
	Associate’s/technical degree		197 (22.62)	298 (23.19)	1309 (19.73)	
	Bachelor’s degree		185 (21.24)	323 (25.14)	2050 (30.90)	
	Post-graduate degree		132 (15.15)	207 (16.11)	1726 (26.01)	
**Annual income, n (%), n=8752**						
	<$15,000		163 (19.24)	153 (11.99)	409 (6.17)	<.001
	$15,000-29,999		163 (19.24)	268 (21.00)	846 (12.76)	
	$30,000-49,999		138 (16.29)	217 (17.01)	1055 (15.91)	
	$50,000-100,000		127 (14.99)	288 (22.57)	1786 (26.94)	
	>$100,000		59 (6.97)	120 (9.40)	1136 (17.14)	
	I do not wish to answer		197 (23.26)	230 (18.03)	1397 (21.07)	
**Health insurance, n (%), n=7977**						
	Private		300 (34.25)	421 (36.90)	3148 (52.82)	<.001
	Public		530 (60.50)	685 (60.04)	2651 (44.48)	
	None		46 (5.25)	35 (3.07)	161 (2.70)	
Current age (years), mean (SD)			61.25 (10.51)	59.58 (10.16)	55.97 (10.18)	<.001
Disease duration (years), mean (SD)			30.38 (12.76)	28.70 (12.27)	24.99 (11.65)	<.001
**Patient Determined Disease Steps, n (%), n=8844**						
	Mild		216 (23.05)	347 (27.17)	2583 (38.96)	<.001
	Moderate		218 (23.27)	337 (26.39)	1746 (26.33)	
	Severe		503 (53.68)	593 (46.44)	2301 (34.71)	
**Cognition, n (%), n=8839**						
	Normal		169 (18.15)	233 (18.17)	1566 (23.63)	<.001
	Mild		455 (48.87)	679 (52.96)	3561 (53.74)	
	Moderate-severe		307 (32.98)	370 (28.86)	1499 (22.62)	

^a^The number of total responses for each characteristic varied as the respondents were not required to answer every question.

**Table 4:** Multivariable logistic regression: characteristics associated with functional health literacy.

Characteristic					METER^a^						NVS^b,c^				
					OR			95% CI		OR				95% CI	
**Gender**	
	Male				1.0					1.0					
	Female				2.26			1.97, 2.59		1.83			1.60, 2.08		
**Age**															
	≤35				1.0					1.0					
	>35 to ≤50				1.52			1.10, 2.11		0.99			0.65, 1.53		
	>50 to ≤65				1.78			1.30, 2.44		0.61			0.40, 0.92		
	>65				1.66			1.18, 2.33		0.36			0.24, 0.56		
**Education**															
	High school diploma or less				1.0					1.0					
	Associate’s/technical degree				1.08			0.92, 1.26		1.88			1.58, 2.24		
	Bachelor’s degree				1.82			1.55, 2.14		1.67			1.43, 1.95		
	Post-graduate degree				2.72			1.24, 3.31		1.19			1.02, 1.40		
**Annual income**															
	<$15,000				1.0					1.0					
	$15,000-29,999				1.33			1.05, 1.68		1.45			1.16, 1.81		
	$30,000-49,999				1.40			1.10, 1.76		2.13			1.70, 2.66		
	$50,000-100,000				1.76			1.40, 2.21		2.45			1.98, 3.04		
	>$100,000				2.38			1.81, 3.12		3.02			2.33, 3.91		
	I do not wish to answer				1.47			1.17, 1.85		2.06			1.66, 2.55		
**Cognition**															
	Normal				1.0					1.0					
	Mild				0.94			0.80, 1.11		0.86			0.74, 1.01		
	Moderate-severe				0.80			0.67, 0.96		0.67			0.56, 0.80		
**Patient Determined Disease Steps**															
	Mild			-						1.0					
	Moderate									0.95			0.81, 1.11		
	Severe									0.73			0.63, 0.84		

^a^c-statistic = 0.67; Hosmer Lemeshow Goodness of Fit χ^2^_8_ =5.4, *P*=.72

^b^c-statistic = 0.70; Hosmer Lemeshow Goodness of fit χ^2^_8_=13.5, *P*=.09

^c^NVS = Newest Vital Sign

### Comorbidities and Health Behaviors

In total, 6973 (78.05%) of 8934 participants reported one or more comorbid conditions with 2868 (32.1%) reporting hypertension, 3328 (37.25%) depression, 2804 (31.39%) hyperlipidemia, 1413 (15.82%) migraine, 1152 (12.89%) autoimmune thyroid disease, and 1032 (11.55%) reporting cancer. The remaining comorbidities were reported by fewer than 10% of respondents. Most respondents did not smoke currently (7708/8811, 87.48%) and 3061/8797 (34.78%) denied any alcohol consumption. The mean (SD) BMI of respondents was 26.93 (6.48), with 2625/8741 (30.03%) being overweight and 2239/8741 (25.61%) being obese.

The proportion of respondents with any comorbidity was slightly higher among those with greater health literacy on the METER (Z=-1.81, *P*=.07 for linear trend) and on the NVS (Z=-5.57, *P*<.001 for linear trend). Respondents who reported being nonsmokers were more likely to have functional literacy on the METER and adequate literacy on the NVS than smokers (both *P*<.001, Multimedia Appendix 1). However, the frequency of any alcohol consumption was higher among respondents with higher health literacy than among those with lower health literacy as measured by the METER and the NVS (both *P*<.001, Multimedia Appendix 1). Overweight and obesity were more common among those with lower health literacy (METER *P*=.006; NVS *P*=.007).

### Health Care Utilization

During 6 months prior to survey administration, 1275/8807 (14.48%) respondents presented to an emergency room and 831/8792 (9.45%) were hospitalized. Participants were less likely to report an ER visit (*P*=.002) or hospitalization (*P*<.001) if they had higher literacy on the METER (Multimedia Appendix 1, Figure 1A). Similarly, participants were less likely to report an ER visit or hospitalization if they had adequate literacy on the NVS (both *P*<.001, Multimedia Appendix 1, Figure 1B).

In an unadjusted logistic regression model, low literacy on the METER was associated with 28% increased odds of any ER visit (OR 1.28; 95% CI 1.11-1.48). In a multivariable logistic regression model adjusting for income, disability, and cognitive impairment, low literacy on the METER was associated with 13% increased odds of any ER visit (OR 1.13; 95% CI 0.96-1.33) (Table 5).

**Table 5:** Association of health literacy assessed by the Medical Term Recognition Test (METER) with emergency room (ER) visits and hospitalizations.

Characteristic				ER visits^a^				Hospitalizations^b^			
				OR		95% CI		OR		95% CI	
**METER**	
	Functional literacy			1.0				1.0			
	Low literacy			1.13		0.96, 1.33		1.19		0.98, 1.44	
**Gender**											
	Male							1.0			
	Female							0.75		0.62, 0.90	
**Annual income**											
	<$15,000			1.0				1.0			
	$15,000-29,999			0.65		0.51, 0.84		0.72		0.53, 0.97	
	$30,000-49,999			0.56		0.44, 0.72		0.63		0.47, 0.85	
	$50,000-100,000			0.50		0.40, 0.63		0.52		0.39, 0.69	
	>$100,000			0.48		0.37, 0.63		0.51		0.36, 0.72	
	I do not wish to answer			0.44		0.34, 0.56		0.57		0.43, 0.77	
**Cognition**											
	Normal			1.0				1.0			
	Mild			1.18		0.99, 1.42		1.26		1.01, 1.58	
	Moderate-severe			1.73		1.42, 2.12		1.62		1.27, 2.07	
**Patient Determined Disease Steps**											
	Mild			1.0				1.0			
	Moderate			1.40		1.17, 1.68		1.33		1.03, 1.71	
	Severe			1.92		1.63, 2.26		2.93		2.37, 3.62	

^a^c-statistic = 0.64; Hosmer Lemeshow Goodness of fit χ^2^_8_ = 2.96 *P*=.89

^b^c-statistic = 0.68; Hosmer Lemeshow Goodness of fit χ^2^_8_ =10.7 *P*=.22

In an unadjusted logistic regression model, low literacy on the NVS was associated with 58% increased odds of any ER visit (OR 1.58; 95% CI 1.39-1.79). In a multivariable model adjusting for income, disability, and cognitive impairment, low literacy on the NVS was still associated with increased odds of any ER visit (OR 1.28; 95% CI 1.10-1.48) (Table 6).

In an unadjusted logistic regression model, low literacy on the METER was associated with 28% increased odds of any overnight hospitalization (OR 1.28; 95% CI 1.11-1.48). In a multivariable logistic regression model adjusting for gender, income, disability and cognitive impairment, low literacy on the METER was associated with 19% increased odds of any hospitalization (OR 1.19; 95% CI 0.98-1.44) (Table 5).

In an unadjusted logistic regression model, low literacy on the NVS was associated with 58% increased odds of any overnight hospitalization (OR 1.58; 95% CI 1.36-1.85). In a multivariable logistic regression model, low literacy on the NVS was associated with 17% increased odds of any hospitalization (OR 1.17; 95% CI 0.97-1.40) (Table 6).

**Table 6:** Association of health literacy (NVS) with emergency room (ER) visits and hospitalizations.

Characteristic				ER visits^a^				Hospitalizations^b^			
				OR		95% CI		OR		95% CI	
**NVS**	
	Functional literacy			1.0				1.0			
	Low literacy			1.28		1.10, 1.48		1.17		0.97, 1.40	
**Gender**											
	Male							1.0			
	Female							0.72		0.60, 0.87	
**Annual income**											
	<$15,000			1.0				1.0			
	$15,000-29,999			0.68		0.53, 0.87		0.77		0.57, 1.04	
	$30,000-49,999			0.59		0.46, 0.75		0.67		0.50, 0.91	
	$50,000-100,000			0.53		0.42, 0.66		0.54		0.40, 0.72	
	>$100,000			0.50		0.38, 0.65		0.51		0.36, 0.73	
	I do not wish to answer			0.46		0.36, 0.59		0.60		0.45, 0.81	
**Cognition**											
	Normal			1.0				1.0			
	Mild			1.17		0.98, 1.41		1.25		0.99, 1.57	
	Moderate-severe			1.73		1.42, 2.11		1.63		1.27, 2.09	
**Patient Determined Disease Steps**											
	Mild			1.0				1.0			
	Moderate			1.37		1.14, 1.64		1.31		1.02, 1.69	
	Severe			1.86		1.58, 2.20		2.97		2.39, 3.68	

^a^c-statistic = 0.64; Hosmer Lemeshow Goodness of Fit χ^2^_8_=12.9, *P*=.12

^b^c-statistic = 0.68; Hosmer Lemeshow Goodness of Fit χ^2^_8_=6.67, *P*=.57

## Discussion

### Principal Results

We investigated health literacy in a sociodemographically diverse population of persons with MS. We found that 65.52% of respondents had functional health literacy on both the METER and the NVS. Furthermore, functional literacy was associated with greater comfort and perceived skill at using electronic health information as assessed using eHEALS. Although most respondents performed well on the METER and the NVS instruments, lower health literacy was associated with an increased risk of smoking, overweight and obesity, comorbidity, visits to the emergency room and overnight hospitalizations.

As assessed by the METER, 81% of the NARCOMS population had functional literacy while nearly 75% had adequate literacy as assessed by the NVS; the latter instrument has a greater emphasis on numeracy. We were unable to identify other studies which have evaluated this issue in MS. Findings varied in other chronic diseases. Approximately 70% of individuals diagnosed with COPD have functional health literacy [31], as compared to 88% in persons with rheumatoid arthritis, and 82.5-85% in persons with heart disease [3,32].

Sociodemographic characteristics associated with greater odds of having functional health literacy included females, older age, higher socioeconomic status, normal self-reported cognition, and lower levels of disability. The association of socioeconomic status and health literacy is consistent across populations, including those with heart failure [3], chronic obstructive pulmonary disease [31], and rheumatoid arthritis [33], among others. In some populations older age is associated with lower rather than higher health literacy, and the association of age with health literacy also varies with the instrument used [3,33]. As we found in our population, lower health literacy is associated with worse health status [31]. This is a complicated issue to understand in MS where cognitive impairment may develop over the course of the disease and could lead to declines in health literacy. Longitudinal studies will be needed to determine the directionality of these relationships in MS.

Lower health literacy was associated with a greater frequency of smoking and obesity, but a lower frequency of regular alcohol use and comorbidity. Findings in other populations regarding these associations have been inconsistent [7]. Our findings may reflect unmeasured confounders, differential health behaviors according to health literacy, or differential reporting according to health literacy. Persons with lower health literacy have less knowledge of chronic diseases, and we speculate that they may not report comorbidity as accurately. These findings will require further evaluation in future studies. Respondents who did not have functional health literacy had increased odds of emergency room visits, after accounting for potential confounders. This association was stronger for the NVS (OR 1.28; 95% CI 1.10-1.48), which captures numeracy, than for the METER (OR 1.13; 95% CI 0.96-1.33). Similarly, respondents who did not have functional health literacy had increased odds of hospitalization although these associations were marginally nonsignificant for the METER (OR 1.19; 95% CI 0.98-1.44) and the NVS (OR 1.17; 95% CI 0.97-1.40). Lower health literacy is consistently associated with greater health care utilization in other populations, although the magnitude of the association varies across populations and outcomes studied [7].

### Limitations

The response rate was 69.27% and responders were more likely to be white and to have a higher annual income; thus, our findings may not be applicable to nonwhites and those of lower socioeconomic status. The NARCOMS population does not fully represent the MS population in the United States, but its characteristics are similar to those reported for other MS populations [34,35]. Furthermore, it is a large, sociodemographically diverse population comprised of participants who receive care in community-based and academic centers. The NVS was not designed to be self-administered; however, it has been successfully self-administered in other studies [36]. None of the health literacy measures used was ideal, and the literature does not provide a clear understanding of the relationships between them. In their critical appraisal of health literacy tools, Jordan et al found that no existing instrument fully measured health literacy with respect to the person’s ability to seek, understand, and use health information [24]. Moreover, construct validity was variable and the sensitivity to change of most instruments has not been evaluated. These challenges are supported by the relatively weak correlations among the three instruments used in this study. At least one study has raised concerns about the validity of the eHEALS due to low correlations with internet use [37]. Although we included a measure of cognition, it is unlikely that this fully accounted for cognitive impairment, given the complex relationships between subjective and objective measures of cognition [38]. Because this was an initial study evaluating health literacy, the design was cross-sectional, limiting our ability to assess causal relationships between health literacy and the outcomes of interest.

### Conclusions

Health literacy is under-studied in MS. Our findings suggest that it is associated with adverse health behaviors, and increased health care utilization. Future work should seek to develop better methods of defining and assessing health literacy in MS population, confirm these findings, elucidate causal pathways, examine a broader range of health outcomes including adherence to therapy, and ultimately, evaluate the impact of interventions aimed at improving health literacy in the MS population.

## Multimedia Appendix 1

Frequency of health behaviours and health care utilization according to health literacy scores.

## Abbreviations

BMI: body mass index
CI: confidence interval
eHEALS: eHealth Literacy Scale
ER: emergency room visits
METER: Medical Term Recognition Test
MS: multiple sclerosis
NARCOMS: North American Research Committee on Multiple Sclerosis Registry
NVS: Newest Vital Sign
OR: odds ratio
PCA: principal components analysis
PDDS: Patient Determined Disease Steps
SD: standard deviation
