# Supplementary material for: Health Literacy Association With Health Behaviors and Health Care Utilization in Multiple Sclerosis: A Cross-Sectional Study
Source: Interact J Med Res. 2014 Feb 10;3(1):e3. doi: 10.2196/ijmr.2993 (PMC3936300; doi:10.2196/ijmr.2993)

Figure 1A. Frequency of health behaviours and health care utilization according to health literacy scores on the METER

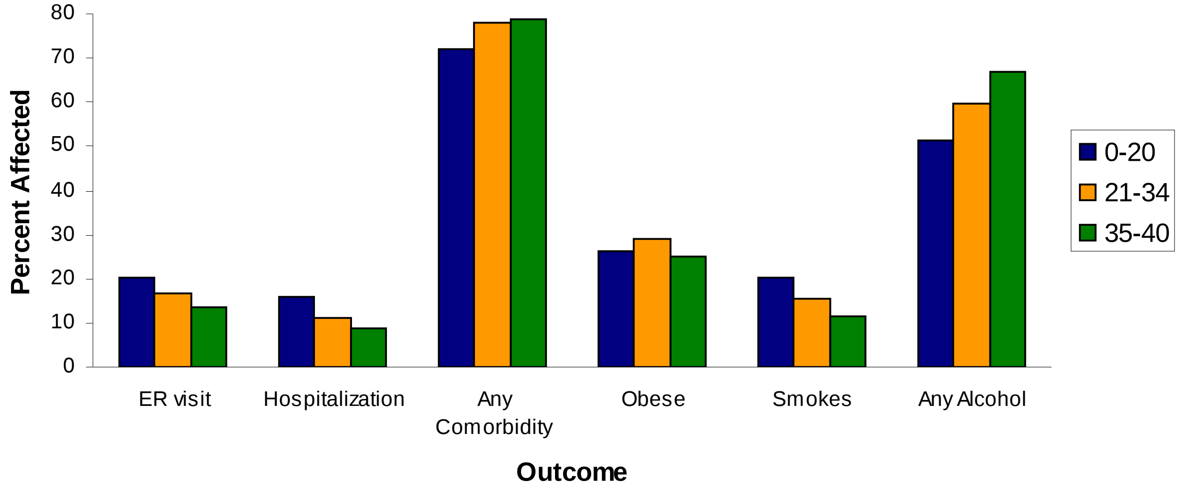

Figure 1B. Frequency of health behaviours and health care utilization according to health literacy on the Newest Vital Sign

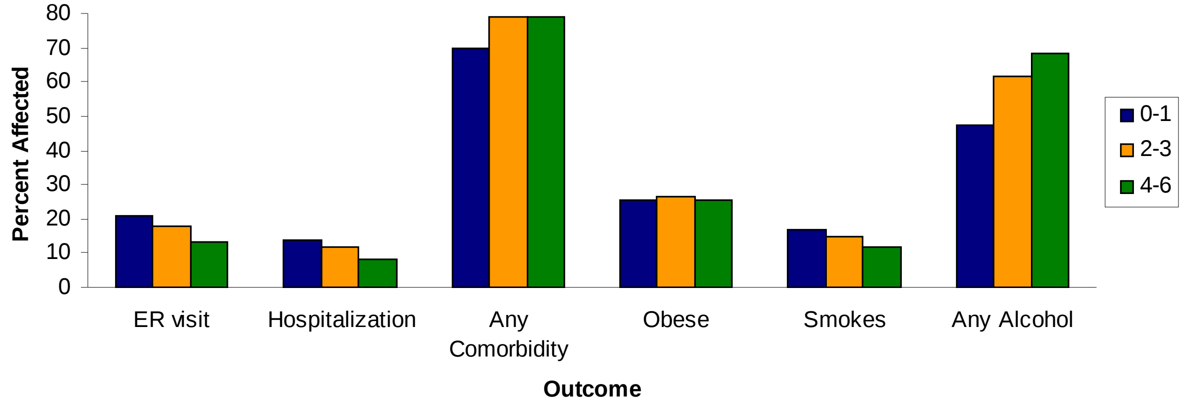

Supplement: Supplementary file 1 [file ijmr_v3i1e3_app1.pdf]
